# The impact of cognitive training in substance use disorder: the effect of working memory training on impulse control in methamphetamine users

**DOI:** 10.1007/s00213-017-4597-6

**Published:** 2017-03-21

**Authors:** Samantha J. Brooks, L Wiemerslage, KH Burch, SA Maiorana, E Cocolas, HB Schiöth, K Kamaloodien, DJ Stein

**Affiliations:** 1 UCT Department of Psychiatry and Mental Health, Groote Schuur Hospital, Anzio Road, Observatory, Cape Town, South Africa; 2 Department of Neuroscience, Uppsala University, Uppsala, Sweden; 3 Department of Neuroscience, University of Nottingham, Nottingham, UK; 4 UCT Department of Psychology, Cape Town, South Africa; 5 Department of Psychology, University of the Western Cape, Cape Town, South Africa; 6 MRC Unit on Anxiety and Stress Disorders, Cape Town, South Africa

**Keywords:** Working memory, Impulsivity, Self-regulation, Methamphetamine

## Abstract

**Objectives:**

Impulsivity is a vulnerability trait for poor self-regulation in substance use disorder (SUD). Working memory (WM) training improves impulsivity and self-regulation in psychiatric disorders. Here we test WM training in methamphetamine use disorder (MUD).

**Methods:**

There are 15 MUD patients receiving inpatient treatment as usual (TAU) and 20 who additionally completed WM cognitive training (CT) and 25 healthy controls (HC). MANCOVA repeated measures analyses examined changes in impulsivity and self-regulation at baseline and after 4 weeks.

**Results:**

Post hoc *t* tests confirmed that at baseline, feelings of self-control were significantly lower in the MUD (*t* = 2.001, *p* = 0.05) and depression was higher (*t* = 4.980, *p* = 0.001), as was BIS total impulsivity (*t* = 5.370, *p* = 0.001) compared to the HC group. Total self-regulation score was higher in HC than MUD patients (*t* = 5.370, *p* = 0.001). CT had a 35% learning rate (*R* ^2^ = 0.3523, *p* < 0.05). Compared to follow-up TAU, follow-up CT group had higher self-reported mood scores (*t* = 2.784, *p* = 0.01) and higher compared to CT baseline (*t* = 2.386, *p* = 0.036). Feelings of self-control were higher in CT than TAU at follow-up (*t* = 2.736, *p* = 0.012) and also compared to CT baseline (*t* = 3.390, *p* = 0.006), lack of planning significantly improved in CT between baseline and follow-up (*t* = 2.219, *p* = 0.048), as did total impulsivity scores (*t* = 2.085, *p* = 0.048). Measures of self-regulation were improved in the CT group compared to TAU at follow-up, in total score (*t* = 2.442, *p* = 0.038), receiving score (*t* = 2.314, *p* = 0.029) and searching score (*t* = 2.362, *p* = 0.027). Implementing self-regulation was higher in the CT group compared to TAU (*t* = 2.373, *p* = 0.026).

**Conclusions:**

WM training may improve control of impulsivity and self-regulation in people with MUD.

## Introduction

Impulse control disorder is considered to be a characteristic trait of a variety of psychiatric conditions, in particular those where failure to resist drives or temptations to perform acts become harmful to sufferers and to others (Atmaca 2014). Impulsivity encompasses ‘knee-jerk’ behaviours that are associated with choosing an immediate over a delayed reward (Hoffman et al. 2006), risky decision-making (Duarte et al. 2012), memory impairment and higher levels of depression (Casaletto et al. 2015). Substance use disorder (SUD) is one example of a psychiatric condition that is characterised by deficits in impulse control, as well as alterations in dopaminergic reward pathways in the brain, which has been substantiated by a large meta-analysis of 97 studies (Smith et al. 2014). More specifically, methamphetamine use disorder (MUD) is the most prevalent SUD in South African (Plüddemann and Parry 2012) and is associated with impulsive behaviours and deficits in executive functioning that may underlie the South African pandemic of HIV and risky sexual behaviour and other neuropsychological deficits associated with social problems (Weber et al. 2012; Marquine et al. 2014; Durvasula and Hinkin 2006). Impulsive behaviour, while perhaps exacerbated by MUD for example, is also suggested to be an endophenotypic trait—behaviour derived from genetic susceptibility that predicts vulnerability for compulsive drug taking (Belin et al. 2015), as well as altered brain processes that underscore a higher likelihood of relapse after a course of treatment (Everitt 2014).

Given that impulsivity appears to be a trait central to vulnerability and persistence of relapse after standard treatment in those with SUD (Adinoff et al. 2016), it is pertinent to consider adjuncts to treatment that aim to improve brain processes associated with impulse control and self-regulation. Currently, standard psychological interventions for SUD are founded in cognitive behavioural therapy (CBT), which target affect, behaviour and cognitions (A-B-C) pertaining to perceptions about self, the world and others (Magill and Ray 2009). However, adjunctive treatment that aims to encourage inherent neural plasticity with repetitive and increasingly difficult cognitive training (Keshavan et al. 2014) may improve decision-making and self-regulation and therefore the prognosis for relapse in those with SUD. For example, the executive function working memory (WM) is a dynamic neural process associated with decision-making and improved self-regulation of cognitive-affective states, and people with SUD are known to be most susceptible to dysfunction in WM processes (Bickel et al. 2014). Furthermore, WM training targets cortico-limbic neural systems associated with cognitive control that is damaged in those with SUD (Brooks 2016, Brooks et al. 2016).

In order to test the effects of WM training, particularly in people with MUD, which can be regarded as the most potent and prevalent drug of abuse in South Africa, associated with the contraction and spread of HIV (Plüddemann and Parry 2012), we have recently developed a smartphone-based WM training intervention in Cape Town, South Africa (Brooks et al. 2016), to reach out to the need for a low-cost adjunct to treatment that can target populations whose access to standard treatment is strained but whose access to a smartphone is not (Anthes 2016). Moreover, we have demonstrated that daily patient engagement in our smartphone-based N-back WM task is easy for clinicians to implement as part of their standard treatment programme for SUD. For example, the patients sit in a classroom and complete a 15-min session of our smartphone intervention twice daily, sending scores back to researchers/clinicians for tracking. During these sessions, patients are required to quietly attend to the task without disruption and touch the screen of their phone when they see the target letter in a series of letters (see “Materials and methods” for more detailed explanation). In this vein, WM training has been effectively utilised to improve prognosis for other psychiatric populations, particularly in disorders that are comorbid with SUD (Akindipe et al. 2014), such as learning difficulties (Peijnenborgh et al. 2016), mood disorders (Meusel et al. 2013), psychosis (Li et al. 2015) and anxiety (Sari et al. 2016). In terms of efficacy of WM training for SUD, it has shown to be an effective strategy to reduce alcohol use by increasing control over automatic impulses to drink alcohol (Houben et al. 2011) and to reduce engagement in stimulant use (Bickel et al. 2011). Furthermore, if some cases of obesity are regarded as a form of *food addiction*, then complementary findings using WM training suggest improvements in weight control (Verbeken et al. 2013). However, WM training, while modestly improving cognitive performance in smokers, does not appear to alter smoking cessation rates (Loughead et al. 2016), and so WM training may be beneficial to some, but not all SUD patients.

Against this background, no study has yet measured the effects of WM training on impulse control in MUD, which may have differential effects on impulse control compared to other drugs of abuse such as cocaine (Bickel et al. 2011). As such, WM training may or may not be effective for MUD. Nevertheless, given that WM training is effective for those who abuse other stimulants (Bickel et al. 2011), and that we have recently shown brain changes in those with MUD linked to changes in impulsivity scores (Brooks et al. 2016), here we hypothesise that the addition of daily WM cognitive training alongside treatment as usual (TAU) for patients with MUD will be associated with improvements on a range of self-report measures of impulsivity and self-regulation in patients being treated for MA dependence.

## Materials and methods

### Participants

**Fig. 1:**
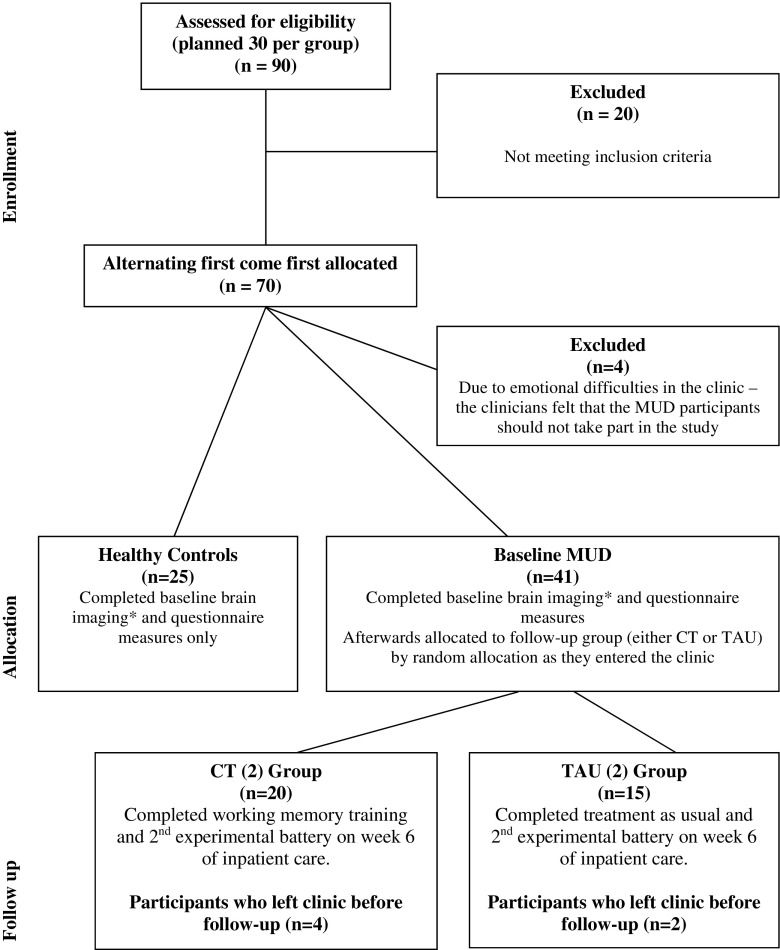
CONSORT diagram to describe how healthy controls (HC) as well as methamphetamine use disorder (MUD) participants were recruited to either the treatment as usual (TAU) group or the cognitive training (CT) group. (*Asterisk*) Brain scanning data (structural and functional magnetic resonance imaging) was also collected at baseline and follow-up in HC, MUD, TAU and CT groups, and this data is published elsewhere (Brooks et al. 2016) with further data currently in preparation

See Fig. 1 for CONSORT (Consolidated Standards of Reporting Trials) recruitment diagram. Sixty MUD individuals (confirmed to be MUD prior to clinical admittance, abstinence was confirmed by clinical screening procedures and enforced during the clinical program) and 30 healthy controls (HC) aged between 18 and 50 were initially invited to be screened to take part in the study, at a local rehabilitation clinic in Cape Town, South Africa, and at the research offices between January 2013 and September 2014. At the end of the study, 35 MUD in-patients were included in data analysis (*n* = 7 did not meet the inclusion criteria, *n* = 8 could not be scanned in time for follow-up due to scanner closures, *n* = 4 were excluded due to emotional difficulties during treatment as usual and *n* = 6 patients dropped out and returned home before the end of the experiment/programme). The mean duration of MUD exposure prior to admittance to the clinic for the remaining participants who completed the study over 4 weeks was 9.5 years (s.d. = 3.65). According to clinicians, all patients in the study were abstinent from drug use for at least 2 weeks before being randomly assigned to one of two groups. After baseline questionnaire measures were completed, the MUD baseline participant was given either (a) rehabilitative TAU (*n* = 15) or (b) in addition to TAU 4 weeks of cognitive training (CT; *n* = 20) using a WM task. The study was approved by the University of Cape Town Human Research Ethics Committee (ref: 554/2012) and adhered to the guidelines set out in the Declaration of Helsinki. This was a pilot, exploratory study to examine the effects of WM training on self-report measures, not clinical outcomes, and was therefore not a clinical trial or intervention.

Inclusion criteria for the MUD group are as follows: (a) methamphetamine was the main substance of use; (b) no DSM history of abusive alcohol use (excluding infrequent alcohol use or concomitant cannabis/methaqualone use); (c) no current or previous history of psychosis as confirmed by clinical staff and screening questionnaires; (d) no prescribed medication during the study (e.g. anti-psychotic, anti-depressant, anti-anxiety medications and/or medications for attention deficit hyperactivity disorders that may alter cognitive performance and would be a potential confounding factor that may alter the effects of WM training [Barch 2004]); (e) negative HIV status; and (f) fluent in English. Inclusion criteria for the HC group are as follows: (a) no DSM history of abusive alcohol use (excluding infrequent alcohol use or concomitant cannabis/methaqualone use); (b) no current or previous history of psychiatric disorder (including clinical anxiety, depression and occasional drug use) as confirmed by screening questionnaires; (c) no prescribed medication during the study; (d) negative HIV status; and (e) fluent in English.

All participants were compensated with 150 ZAR (South African currency “Rands” equivalent to approximately $15) in the form of a supermarket voucher for baseline participation and 150 ZAR for participation at follow-up.

### Clinical setting

MUD patients were recruited from an in-patient rehabilitation clinic in Cape Town that houses a maximum of 40 male and female patients at any given time. The rehabilitation program runs for a total of 8 weeks, during which time patients are provided with six meals a day (up to 3500 cal), consisting of a large breakfast, lunch and supper with three small snacks in between. TAU involves 1-h daily sessions of dialectical behavioural therapy (DBT) from Monday to Friday for 6 weeks, a total of 30 1-h sessions. DBT, a form of cognitive behavioural therapy (CBT), addresses maladaptive affective responses and has proven successful in treating SUD. In addition to DBT, patients attended psycho-education sessions addressing basic and social skills development and physical activities outside. Of note, the first 2 weeks of the program were regarded as the induction period and so researchers were not permitted to interview or contact the patients during this time. Thereafter, researchers were given 4 weeks to conduct data collection; for TAU, this consisted of baseline and follow-up questionnaires on 2 days; additionally for CT, this consisted of daily half-hour cognitive training (excluding weekends). The final 2 weeks of the 8-week programme were devoted to preparing patients for re-entry to the outside world (and so researchers were again not permitted to contact patients during this time).

In addition to TAU, during the 4-week data collection period, the CT group received training in a classroom at the clinic, using a computer-based WM task called “Curb Your Addiction (C-Ya)” (for details see http://www.drsamanthabrooks.com/curb-your-addiction) that was developed by the authors with Fontera Digital Works (www.fontera.com). Free copies of the software are available upon request. C-Ya is a modified version of the N-back task (the modification being a distracting peripheral mosaic to mimic peripheral distraction in real life), ranging from 0-back to 3-back. The N-back task was originally introduced by Kirchner (Kirchner 1958) and requires a response to a specified target letter as single letters appear on the screen consecutively. In the present study, the letter ‘X’ was the target for ‘0-back’; for ‘1-back’ when the current letter was the same as the ‘1 before’; for ‘2-back’ when the current letter was the same as ‘2 before’ and ‘3 before’ for ‘3-back’. Patients identified targets by pressing the space bar on the computer keyboard. During the standard version of the C-Ya task, participants began by completing 30 min of 0-back and they progress the next day on to the consecutively higher level after achieving at least 80% accuracy on the prior level. An 80% threshold was used in relation to previous data demonstrating the effects of WM training on neural function; during the previous study, the highest level of accuracy attained was 80%. Therefore, we decided to use this as a guideline for our study (Olesen et al. 2003). Accuracy was calculated using the following algorithm:

[1 − ((number of commissions + number of omissions)/total possible correct)] × 100 (Miller et al. 2009), where commissions were responses to non-target letters; omissions were failures to respond to a target, and total possible correct was the total target letters.

Participants were not permitted to progress on to the consecutively higher level during the training until they achieved at least 80% accuracy on the previous level, and due to this, the task is considered to be adaptive (Keshavan et al. 2014). Participants in this study were required to engage in the task five times a week for 4 weeks (maximum 20 sessions). We calculated the learning rate only in the most difficult 3-back level, as the previous levels (0-back, 1-back and 2-back had ceiling effects and limited variance in performance and were completed during the first induction week of the 4-week training period). Learning rate was calculated using Wright’s learning curve equation (Wright 1936):

$$ Y={aX}^b, $$

*Y*: the cumulative average time (or cost) per unit
*a*: time (or cost) required to produce the first unit
*X*: the cumulative number of units produced
^*b*^: slope of the function when plotted on log-log paper
=: log of the learning rate/log of 2

We report the learning rate with the regression coefficient, but only in the participants who, while continuously engaging in the working memory training, were able to maintain a consistent 3-back performance in the final stages of their training.

### Clinical measures

#### Interview with clinical caseworker at the treatment centre

In the first week, all MUD patients underwent a routine one-to-one interview with a qualified clinician to ascertain diagnosis and comorbidities, confirming MUD and whether to prescribe medication. Based on the information from clinical interviews and after permission from the clinical staff, we recruited non-medicated, non-psychotic methamphetamine-dependent, HIV-negative, currently abstinent in-patients most likely to complete the full 8-week programme at the clinic.

#### Structured Clinical Interview for Diagnosis of Axis I DSM-IV disorders (First et al. 2002) patient version with psychotic screen and non-patient version

We selected patients who were identified by clinical staff to attend an interview with a researcher using the SCID for DSM-IV, which was conducted at the clinic by a qualified research scientist. For the HC group, the SCID was conducted at the university research offices. The SCID included screening questions for substance abuse (including alcohol and other drugs), mood, thought, anxiety and general screening questions. In addition to this, we asked participants to complete the following self-report measures.

### Self-report measures

#### Hospital Anxiety and Depression Scale (Zigmond and Snaith 1983)

Hospital Anxiety and Depression Scale (HADS) is a 14-item questionnaire used to assess patients’ levels of anxiety and depression. Seven of the items relate to depression and 7 to anxiety. Items are rated on a 4-point scale, with a maximum score of 21 for both anxiety and depression. A score of 0–7 is ‘normal’, 8–10 is considered ‘borderline’ and 11 or higher is considered significant.

#### Barratt Impulsivity Scale (Patton et al. 1995)

Barratt Impulsivity Scale (BIS) is a 30-item questionnaire designed to assess individual levels of impulsivity. Items are scored on a four-point scale (rarely/never, occasionally, often, almost always/always) to give six first-order factors (attention, motor, self-control, cognitive complexity, perseverance and cognitive instability) and 3-s order factors (attentional, motor and non-planning) and a total impulsivity score.

#### Self-Regulation Questionnaire (Brown et al. 1999)

The Self-Regulation Questionnaire (SRQ) is a 63-item questionnaire designed to assess an individual’s self-regulatory processes, measuring seven factors of self-regulation: (a) receiving relevant information, (b) evaluating information and comparing it to norms, (c) triggering change, (d) searching for options, (e) formatting a plan, (f) implementing the plan and (g) assessing the plan’s effectiveness. Items are scored on a five-point scale (strongly disagree, disagree, unsure, agree, strongly agree) and participants are asked to respond based on how well each statement describes them. It has been verified to give good internal consistency and reliability in a sample of young adults, particularly the total score.

#### Trail Making Test (Tombaugh 2004)

The Trail Making Test (TMT) is a paper-based neuropsychological measure of an individual’s speed of processing, mental flexibility, executive function (e.g. working memory), visual searching and scanning abilities. The TMT consists of two parts: TMT-A and TMT-B. TMT-A requires participants to draw a line between 25 numbers evenly distributed on a piece of paper. TMT-B instead requires participants to alternatively join numbers with letters (e.g. 1, A, 2, B, 3, C). The time taken to complete the task and the number of errors are recorded. To account for dexterity, the results from TMT-A are subtracted from the results of TMT-B to produce a final score.

#### Visual Analogue Scale (Reips and Funke 2008)

The Visual Analogue Scale (VAS) is a psychometric response scale, used to assess subjective feelings. In this study, level of happiness (mood), desire for drug and feelings of self-control were assessed. Participants responded by placing a mark on a horizontal line to indicate their current feelings. The left end point of the line represents low happiness/mood, no desire for drug and no feelings of self-control, and the right end point represents high level of happiness/mood, high desire for drug and high feelings of self-control, respectively. The position of the mark on the line was measured and transformed into a percentage for analysis purposes.

### Statistical analyses

Normal distribution and homogeneity of variance was assessed using the Shapiro-Wilk test. A 2 × 2 multivariate analyses of covariance (MANCOVA), with age and education as covariates of no interest, was first conducted between group (HC, baseline MUD) and primary outcome measures: (a) impulsivity (BIS total, VAS desire for drug), (b) self-regulation (SRQ total, VAS feeling of self-control), and (c) executive functioning (TMT). We additionally examined anxiety and depression (HADS, VAS mood) as a secondary outcome measure, given that rates are typically high in patients with MUD vs. HC prior to treatment. The rationale for conducting this first MANCOVA analysis was to establish that there were indeed differences in impulsivity, executive function and self-regulation scores, as well as mood scores, between baseline patients and healthy controls.

We conducted a second repeated measures (according to time) MANCOVA analysis with age and education as covariates of no interest, weighted for duration of drug taking and assessed significant differences between group (TAU and CT) and time (baseline, follow-up) on the primary and secondary outcome measures as above. We chose to include these measures in our model in order to control for the possibility that mood (VAS, HADS scores) and executive function differences (TMT) would influence the effects of TAU vs. CT on changes in impulsivity and self-regulation scores. Additionally, we corrected all analyses for multiple comparisons using the Bonferroni method. Following our repeated measures MANCOVA analyses, we conducted post hoc *t* tests to confirm any significant differences between and within groups and time points. While total and not subscale scores on the separate questionnaires were entered into the MANCOVA analyses (due to their being less power to detect differences using subscales with scores of smaller ranges), we additionally conducted post hoc *t* tests on the subscale scores as an exploratory measure and report these in the tables.

## Results

### Demographic and psychological data

**Fig. 2:**
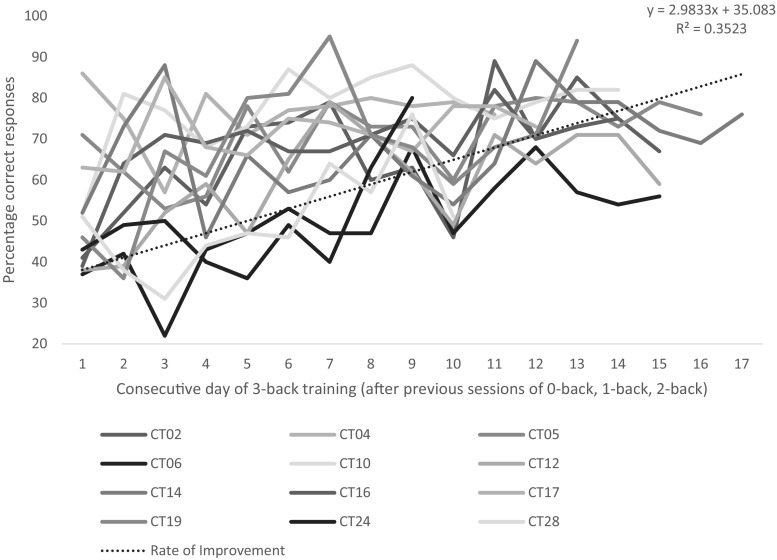
Graph to illustrate the learning rate (according to Wright’s learning curve equation (Wright 1936). *CT* cognitive training; *individual lines* on graph represent individual subjects who were in the CT group; *x axis* number of days engaging in 3-back, *y axis* percentage correctly identified targets. Of note, only *n* = 12 participants were included in this calculation for consistency, because *n* = 8 participants, while they did engage in 4 weeks of cognitive training, did not consistently engage in 3-back but rather sporadic 2-back and 3-back

See Table 1 for demographic data, Table 2 for mean differences between and within groups (HC, MUD baseline, follow-up TAU and CT) and Table 3 for statistical values. In the CT group, an exponential growth (learning) rate of 35% (R^2^ = 0.3523, *p* < 0.05) was observed using the learning curve calculation (see “Materials and methods”, and Fig. 1). While *n* = 20 MUD patients successfully completed the cognitive training over 4 weeks, *n* = 12 patients were able to sustain a consistent 3-back performance—we chose to plot the data in only those who achieved consistent highest level 3-back training to examine the rate of learning (avoiding the confounding effect of performance drop-out during training). In other words, *n* = 8 participants chose to sporadically return to 2-back level after various attempts of 3-back, yet still engaged in training at the lower level (Fig. 2).

**Table 1:** Demographic data on healthy controls, baseline methamphetamine-using patients, follow-up treatment as usual patients and patients who additionally engaged in cognitive training

	Healthy controls (*n* = 25)	All baseline MUD (*n* = 41)	TAU group (*n* = 15)	CT group (*n* = 20)
Age (mean, s.d.)	27.67 (8.714)	29.10 (6.69)	28.11 (6.01)	29.83 (7.32)
Duration drug taking (years)	–	9.5 (3.63)	10.73 (3.96)	9.42 (4.4)
Ethnicity, *n* (%)				
Black	7 (33)	2 (5)	1 (7)	0 (0)
Mixed race	2 (10)	37 (90)	13 (86)	18 (90)
White	12 (57)	2 (5)	1 (7)	2 (10)
Education, *n* (%)				
No matric	1 (5)	28 (68)	9 (60)	14 (70)
Matric	1 (5)	13 (32)	6 (40)	6 (30)
Undergraduate	12 (57)	0 (0)	0 (0)	0 (0)
Honours	4 (19)	0 (0)	0 (0)	0 (0)
PhD	3 (14)	0 (0)	0 (0)	0 (0)

*s.d.* standard deviation, *MUD* methamphetamine use disorder, *TAU* treatment as usual, *CT* cognitive training

**Table 2:** Psychological variables that were analysed in the MANCOVA

	Means (standard deviation)					
Variables	Healthy controls (*n* = 21)	All baseline MA (*n* = 41)	Baseline TAU (*n* = 17)	Baseline CT (*n* = 24)	Follow-up TAU (*n* = 15)	Follow-up CT (*n* = 20)
VAS mood (%)	64 (13)	59 (30)	57 (36)	62 (27)	60 (29)	82 (19)
VAS desire for drug (%)	4 (6)	19 (24)	20 (28)	20 (22)	17 (17)	14 (22)
VAS feelings of self-control (%)	83 (15)	69 (21)	66 (24)	72 (20)	77 (19)	92 (11)
HADS anxiety	7 (3)	7 (2)	8 (1)	7 (2)	7 (3)	6 (2)
HADS depression	2 (2)	6 (3)	6 (2)	5 (4)	6 (8)	3 (3)
Trail Making response time (B-A)	34 (16)	62 (51)	53 (51)	69 (51)	42 (36)	44 (30)
BIS total	53 (9)	66 (10)	68 (8)	64 (11)	67 (13)	60 (11)
SRQ total	233 (23)	227 (23)	221 (15)	229 (29)	220 (18)	246 (27)

*s.d.* standard deviation, *MUD* methamphetamine use disorder, *TAU* treatment as usual, *CT* cognitive training, *VAS* Visual Analogue Scale, *HADS* Hospital Anxiety and Depression Scale, *BIS* Barratt Impulsivity Score, *SRQ* Self-Regulation Questionnaire

**Table 3:** *T* test analyses between patient groups in the main clinical variables (subscales on questionnaire measures were not examined due to lack of power)

*p* value *T* statistic (Cohen’s *d* effect size)							
	Baseline TAU vs. baseline CT	Follow-up TAU vs. follow-up CT	Baseline TAU vs. follow-up TAU	Baseline CT vs. follow-up CT	Baseline MUD vs. follow-up MUD	Baseline MUD vs. follow-up TAU	Baseline MUD vs. follow-up CT
Mood (%)	0.312 1.037 (0.22)	0.024 2.446 (0.92)	0.347 0.968 (0.97)	0.018 2.546 (0.91)	0.034 2.196 (0.49)	0.465 0.741 (0.04)	0.011 2.689 (0.92)
Desire for drug (%)	0.498 0.691 (0)	0.371 0.915 (0.15)	0.410 0.844 (0.13)	0.069 1.904 (0.27)	0.207 1.283 (0.20)	0.379 0.894 (0.13)	0.251 1.169 (0.15)
Feelings of self-control (%)	0.269 1.138 (0.27)	0.021 2.510 (0.96)	0.166 1.446 (0.57)	0.003 3.315 (1.27)	0.005 2.964 (0.86)	0.170 1.407 (0.4)	0.001 3.616 (0.96)
HADS anxiety	0.075 1.880 (0.75)	0.159 1.464 (0.45)	0.177 1.408 (0.44)	0.188 1.358 (0.31)	0.091 1.730 (0.34)	0.438 0.786 (0.06)	0.052 2.015 (0.45)
HADS depression	0.144 1.520 (0.52)	0.050 2.088 (0.8)	0.386 0.889 (0.13)	0.025 2.406 (0.54)	0.322 1.003 (0.14)	0.185 1.356 (0.33)	0.017 2.523 (0.8)
Trail Making response time (B-A)	0.246 1.195 (0.31)	0.439 0.790 (0.07)	0.231 1.242 (0.27)	0.385 0.885 (0.63)	0.06 1.943 (0.47)	0.140 1.517 (0.47)	0.131 1.549 (0.07)
BIS total	0.216 1.277 (0.37)	0.079 1.851 (0.64)	0.466 0.747 (0.03)	0.048 2.085 (0.44)	0.107 1.650 (0.25)	0.351 0.947 (0.15)	0.056 1.985 (0.64)
SRQ total	0.226 1.249 (0.41)	0.005 3.164 (1.53)	0.239 1.221 (0.37)	0.096 1.735 (0.71)	0.212 1.268 (0.26)	0.140 1.517 (0.51)	0.018 2.496 (1.53)

*TAU* treatment as usual, *CT* cognitive training, *MUD* methamphetamine use disorder, *HADS* Hospital Anxiety and Depression, *BIS* Barratt Impulsivity Scale, *SRQ* Self-Regulation Questionnaire

### MANCOVA and post hoc *t* test analyses

#### HC vs. baseline MUD

MANCOVA analyses revealed a significant interaction between groups (HC, MUD) across the primary outcome measures (*F* = 2.365, *p* = 0.043). Furthermore, examining the individual primary/secondary outcome measures revealed differences between HC and MUD in feelings of self-control (*F* = 4.907, *p* = 0.033), HADS depression (*F* = 8.252, *p* = 0.007), BIS total (*F* = 13.712, *p* = 0.001), and SRQ total (*F* = 4.902, *p* = 0.033).

Post hoc *t* tests confirmed that desire for drug was higher in MUD patients than controls (*t* = 3.298, *p* = 0.002), and that feeling of self-control was lower in MUD patients than controls (*t* = 2.001, *p* = 0.05). Furthermore, depression (but not anxiety) was higher in the MUD group than controls (*t* = 4.980, *p* = 0.001), as was BIS total impulsivity (*t* = 5.370, *p* = 0.001). Total self-regulation score was higher in controls than MUD patients (*t* = 5.370, *p* = 0.001), and patients were slower to complete the Trail Making B executive function task than controls (*t* = 3.815, *p* = 0.001).

#### Baseline MUD vs. follow-up

Repeated measures MANCOVA analyses revealed no significant interaction between group (TAU, CT) and time (baseline, follow-up) (*F* = 1.038, *p* = 0.382). However, examining the between-subjects effects of group and time revealed separate significant main effects in type of participant (TAU, CT) and also time (baseline, follow-up) on the total SRQ (*F* = 3.020, *p* = 0.05 and *F* = 4.732, *p* = 0.033, respectively) and a significant difference between time (baseline, follow-up) on the total BIS (*F* = 5.246, *p* = 0.025).

Post hoc *t* tests revealed a significantly higher percentage mood score (as measured by the VAS) in CT compared to TAU group at follow-up (*t* = 2.784, *p* = 0.01) and significantly higher at follow-up CT compared to baseline CT (*t* = 2.386, *p* = 0.036). Similarly, significantly higher percentage feeling of self-control was observed in the CT compared to the TAU group at follow-up (*t* = 2.736, *p* = 0.012) and also significantly higher at follow-up CT compared to baseline CT (*t* = 3.390, *p* = 0.006). In terms of measures of executive function, a significantly faster time to complete the Trail Making Task (B minus A) was observed in the TAU group at follow-up compared to TAU at baseline (*t* = 2.508, *p* = 0.026), an effect that was not observed in the CT group. Significantly improved sense of lack of self-control (as measured by BIS self-control) was observed at follow-up compared to baseline in both the TAU (*t* = 2.566, *p* = 0.022) and CT (*t* = 2.298, *p* = 0.042) groups. Also demonstrated by the BIS, a significantly lower level of cognitive instability (*t* = 2.722, *p* = 0.012) and second-order (lack of) attention (*t* = 2.199, *p* = 0.037) was observed in the TAU group at follow-up compared to baseline, whereas a significantly improved lack of planning was observed in the CT group at follow-up compared to baseline (*t* = 2.219, *p* = 0.048). Finally, total BIS was significantly better in the follow-up CT compared to the baseline CT group (*t* = 2.085, *p* = 0.048). The TAU group had significantly improved anxiety scores at follow-up compared to baseline (*t* = 0.242, *p* = 0.023). Finally, on the SRQ, a significantly higher total score (*t* = 2.442, *p* = 0.038), receiving score (*t* = 2.314, *p* = 0.029) and searching score (*t* = 2.362, *p* = 0.027) were observed in the CT compared to the TAU group at follow-up. Furthermore, the SRQ planning score was significantly higher in the follow-up compared to baseline TAU group (*t* = 2.227, *p* = 0.046), and finally, SRQ implementing was significantly higher in the CT group compared to the TAU group at baseline (*t* = 2.373, *p* = 0.026).

## Discussion

To our best knowledge, we have conducted the first pilot study in patients already in standard treatment for methamphetamine use disorder (MUD) to examine whether 4 weeks of cognitive training (CT) using a progressively difficult adaptive working memory (WM) task alters patients’ self-reports of impulsivity and self-regulation in relation to a healthy group with no history of MUD. We show that the CT group who engaged in the highest level of training had a learning effect of 35% between baseline and after 4 weeks of WM training, which coincided with changes in self-reported impulsivity and self-regulation scores when comparing patient baseline to follow-up.

Our preliminary findings are in line with previous studies advocating WM training as a useful adjunct to treatment to improve cognitions in psychiatric disorders. For example, a recent meta-analysis in people with intellectual disabilities showed improvements in memory with a small effect size (Danielsson et al. 2015). Another meta-analysis of WM training showed WM improvements in young adults (a demographic most susceptible to engaging in substance use) that were associated with long-term cognitive improvements (Peijnenborgh et al. 2016). Moreover, WM training in children with learning difficulties has been linked to improvements in impulsivity, self-regulation and attention deficits (Klingberg et al. 2005; Re et al. 2015). Another recent large meta-analysis of children and adults both with and without attention deficit hyperactivity disorder (ADHD) has shown WM training to improve levels of inattention in daily life that appears to be sustainable regardless of diagnosis (Spencer-Smith and Klingberg 2015). Furthermore, another meta-analysis has shown that CT is linked to improvements in levels of depression and everyday functioning (Motter et al. 2016), which is important to consider given that depression is a well-known comorbidity with SUD (McKetin et al. 2016).

Considering whether WM training improves levels of impulsivity in patients with SUD specifically—and our preliminary findings on self-reported levels of impulsivity suggest that it might—is in line with previous work showing that WM training reduces delay discounting (a measure of impulsivity or ability to delay gratification) in individuals with SUD (Bickel et al. 2011). However, the previous study mainly included people with cocaine dependence rather than, as in our study, only those with MUD, and the abuse of different substances may damage the brain in different ways. Similarly, other studies of WM training in psychiatric populations that are often comorbid with SUD (Akindipe et al. 2014), such as learning difficulties (Peijnenborgh et al. 2016), mood disorder (Meusel et al. 2013), psychosis (Li et al. 2015) and anxiety (Sari et al. 2016), show improvements to clinical measures post-intervention. Thus, while our findings are small and preliminary, our first-of-its-kind pilot study suggests that WM training is also beneficial to patients in treatment for MUD. Our findings are also in line with thinking in the field that WM training may provide benefit to people in treatment for addiction (Bickel et al. 2014).

In terms of the method by which we delivered WM training, a recent review has summarised how computerised WM training, which was used during this study, has been effective at improving relapse rates for SUD, by providing better accessibility to portable devices, improved information capture and adaptability in individual ability in order to engage WM capacity at the highest level (Bickel et al. 2014). In that respect, we adopted smartphone-based delivery of our computerised WM training, in line with recent reports that in low-middle income countries such as South Africa, smartphones are useful at reaching patient populations whose access to treatment facilities is often hampered, but whose access to a smartphone is not (Anthes 2016). In addition, the effects of WM training for those with SUD are suggested to work by harnessing inherent neuroplasticity (e.g. neurobiological learning processes (Lewis 2016)) associated with improvements in the neuropsychological ability to self-regulate to achieve future goals, to control one’s attention and to protect future goals from interferences such as immediate desires and craving (Hofmann et al. 2012).

Sex and gender difference must also be considered in the context of our data, given that in Cape Town, males are more likely than females to present to treatment services with MUD (Weybright et al. 2016) and that we only measured males during this pilot study. However, in light of the evidence documenting sex differences in those with substance and behavioural addictions (Sanchis-Segura and Becker 2016), the impact of gender differences on the outcome of WM training is of importance. For example, currently abstinent males, and not females, who engage in recreational use of cocaine show differences in neurocognitive functioning, such as poorer attention and greater verbal recognition memory (Rahman and Clarke 2005). Additionally, in terms of behavioural addictions, such as compulsive shopping, women as opposed to men show activation of an avoidance coping mechanism (mood compensation) that is secondary to irrational cognitions, which mediates the link between compulsive buying and psychological distress (Ching et al. 2016). Furthermore, given the evidence that sex and gender differences modulate drug consumption as well as the transition towards drug-promoted pathological states (Sanchis-Segura and Becker 2016), it is conceivable that WM training will influence underlying brain processes differently in men and women. For example, the pharmacological impact of MUD on levels of impulsivity and compulsivity is determined by differences in various sex-related factors, such as neuromodulators (e.g. gonadal hormones) that influence neuroplasticity in relevant neural circuits between males and females (Fattore and Melis 2016). Similarly, given the influences of such sex differences, males and females may respond differently to novel treatments, in that males may respond better to cognitive adjuncts (e.g. WM training) whereas females may respond better to behavioural adjuncts (e.g. exercise regimes) (Carroll and Smethells 2016). Future research should provide adequate statistical power to compare males and females to progress the field of WM training in those with addiction disorders.

This pilot study, the first to examine whether WM training alters self-reported impulsivity and self-regulation scores in those being treated for MA use, has some limitations that deserve emphasis. Firstly, self-report measures alone are not sufficient to gauge changes in impulsivity and self-regulation, and clinician or other objectively rated scales are also needed. For example, the go/no-go and stop signal tasks are often used as objective measures of impulsivity/behavioural inhibition (Smith et al. 2014). It would also have been useful to collect subjective measures of the cognitive training process, to gauge whether the patients felt any benefits of WM training during their daily schedule. Furthermore, given various constraints, we were unable to increase the number of participants measured and unfortunately 11 dropped out during their standard treatment. Moreover, we did not include measures of stress levels throughout the study, which may impede progress during TAU or CT, although scores on the HADS have been shown to be significantly related to clinical stress measures (Luckett et al. 2010; Iani et al. 2014). Another limitation is that we only used one cognitive measure to examine cognitive impairment across the groups (e.g. the TMT), and future studies would do well to conduct more extensive testing at baseline of cognitive function as a potential confound to engagement in cognitive training. Additionally, we did not incorporate a non-training computer-based control session into our paradigm, and so while we can compare TAU to those who also received adjunctive CT, it cannot be fully determined that our preliminary findings are due entirely to the WM training, although our findings are in line with previous research in other cohorts. Previous studies have given training over longer periods and observed more significant results. Nevertheless, our preliminary data of the effects of WM training on self-report measures warrants future exploration in terms of offering training as a formal intervention, particularly as other researchers examining different cohorts with impulse control deficits (e.g. ADHD, alcoholism, cocaine use) have shown positive effects of WM training (Klingberg et al. 2005; Houben et al. 2011; Bickel et al. 2011).

## Conclusions

This pilot study is the first to suggest that 4 weeks of WM training could be a promising adjunct for patients receiving treatment for methamphetamine use disorder. While we did not conduct a formal clinical trial and that we only measured self-reported measures, it may still be the case that WM training when offered as an adjunct to treatment as usual can reduce clinical symptoms of impulsivity and improve self-regulation in methamphetamine users, particularly those most vulnerable to relapse.
